# Sleep disturbance is associated with mild cognitive impairment: a community population-based cross-sectional study

**DOI:** 10.1186/s12889-022-14391-3

**Published:** 2022-11-01

**Authors:** Fan Gao, Shan Wei, Liangjun Dang, Yao Gao, Ling Gao, Suhang Shang, Chen Chen, Kang Huo, Jingyi Wang, Jin Wang, Qiumin Qu

**Affiliations:** 1 Department of Neurology, The First Affiliated Hospital of Xi’an Jiaotong University, 277 West Yanta Rd, 710061 Xi’an, China; 2 Huyi Hospital of Traditional Chinese Medicine, Xi’an, China

**Keywords:** Mild cognitive impairment, Sleep disturbance, Cross-sectional study, Risk factor

## Abstract

**Background:**

Sleep is conducive to the elimination of brain metabolites and the recovery of brain function. However, the relationship between sleep disturbance and Mild Cognitive Impairment is not fully been determined.

**Methods:**

This was a community population-based cross-sectional study. A total of 1,443 participants from a village in the suburbs of Xi’an, China were enrolled in 2017. Sleep quality was evaluated using the Pittsburgh sleep quality index (PSQI), and sleep disturbance was defined as a PSQI score > 5. Mini-Mental State Examination (MMSE) was used to assess cognitive function and Mild Cognitive Impairment(MCI) was defined as the MMSE score less than cutoff values and meets the diagnostic criteria. Univariate and multivariate analyses were used to analyze the relationships between sleep disturbance and MCI.

**Results:**

Among 1,443 subjects, 69(4.78%) had MCI, and 830 (57.52%) had sleep disturbance. In bivariate analysis, MCI was associated with sleep disturbance (ρ = 0.094, *P*<0.001). In the binary logistic regression, MCI was positively associated with the sleep disturbance (OR = 2.027, 95%CI = 1.112–3.698, *P* = 0.021). In the internal constitution of PSQI, MCI was negatively associated with the habitual sleep efficiency (OR = 0.447, 95%CI = 0.299–0.669, *P* < 0.001). Compared with waking up before or at 7 am, waking up after 7 am (OR = 0.555, 95%CI = 0.309–0.995, *P* = 0.048), or 8 am (OR = 0.296, 95%CI = 0.097–0.902, *P =* 0.032) was probably more likely to have normal cognition. However, people who slept more than 8 h a day might be more likely to suffer from MCI (OR = 5.560, 95%CI = 1.419–21.789, *P* = 0.014).

**Conclusion:**

Sleep disturbance is associated with Mild Cognitive Impairment. However, the causal relationship between them is not clear. It needs to be further studied.

## Background

Cognitive impairment is a general term for the loss of memory and other mental abilities, with Alzheimer’s disease (AD) being the most common cause of cognitive impairment [1]. β-amyloid (Aβ) deposition in the brain is the main pathological change of AD, and the imbalance of production and clearance of Aβ is the main reason [2–4]. Mild Cognitive Impairment (MCI) describes a state of cognitive functioning that is below defined norms, yet falls short of dementia in severity, which exists across a cognitive continuum with borders that are difficult to define precisely [5]. Sleep is an important physiological process and is conducive to the elimination of brain metabolites and the recovery of brain function [6–8]. The restoration of brain function during sleep may be due to enhanced clearance of waste products, including Aβ, accumulated in waking moments in the central nervous system [9–11].

In recent years, emerging studies have explored the link between sleep and cognitive impairment, with the advancement of the world population aging. Short naps could mitigate the harmful effects of short sleep duration (the total sum of time spent asleep, regardless of sleep stage) on cognitive function [12]. Sleep quality refers to one’s satisfaction with the sleep experience, integrating aspects of sleep initiation, sleep maintenance, sleep quantity, and refreshment upon awakening. Although poor sleep quality had a negative effect on mood and quality of life, sleep quality was not significantly independently associated with MCI in epilepsy [13]. Obstructive sleep apnea (OSA) is characterized by episodes of a complete (apnea) or partial collapse (hypopnea) of the upper airway with an associated decrease in oxygen saturation or arousal from sleep. Cognitive impairment is highly prevalent in patients referred to sleep clinics for suspected OSA in older males [14]. A polygenic score (PS) is an estimate of an individual’s genetic liability to a trait or disease, calculated according to their genotype profile. Higher PS was linked to longer sleep duration and was also associated with better performance in global cognition, fluid reasoning, speed of processing, and language, but not memory [15]. In the sample of the elderly, mainly African Americans, changes in wakefulness after sleep onset were associated with poor cognitive function [16]. Insomnia was defined as trouble falling and/or staying asleep. This can be short-term to last for a few days to a few weeks and for some, it can be chronic and last for more than a month. Narcolepsy refers to a syndrome clinically characterized by excessive daytime sleepiness and sleep attacks plus one or more auxiliary symptoms including cataplexy, sleep paralysis, and hypnagogic hallucinations. Restless leg syndrome is a disorder that causes an overwhelming urge to move legs, usually associated with unpleasant sensations, often during sleep and relieved by movement. In a cross-sectional study conducted in China, the associations between insomnia, narcolepsy, and cognitive impairment were identified, but the associations between OSA, restless leg syndrome, and cognitive impairment were not supported among community residents [17]. Additionally, associations between Rapid-Eye-Movement or Non-Rapid-Eye-Movement sleep behavior disorder and cognitive impairment were conflicted [18–20].

Existing studies have mainly focused on certain aspects of sleep, such as sleep duration, and habitual sleep efficiency. And the results are varied. Little research has used comprehensive and integrated scales to assess various aspects of sleep quality. Besides, the associations between cognitive impairment and wake-up time were less reported. Assessing sleep by monitoring over several days with devices such as polysomnography, is susceptible to environmental and psychological factors. Therefore, more research is needed to comprehensively evaluate sleep quality over a relatively long period. Moreover, most studies have been carried out in high-risk populations, such as epilepsy, Parkinson’s disease, and Hemodialysis. But because these populations might have symptoms of cognitive impairment and sleep disturbance, there will be probably a confounding factor for exploring the relationship between sleep and cognition. In this study, we investigated the relationships between sleep disturbance and MCI in a community population, after excluding the individuals who suffered from AD and other diseases that may influence cognitive function. And sleep quality was evaluated by using the Pittsburgh sleep quality index (PSQI), which covers many aspects of sleep over a 1-month time interval.

## Methods

### Study Design and Population

Participants were enrolled from a cross-sectional study conducted in a village in Xi’an, China, from January 3 to March 26, 2017. The village was randomly selected by the method of cluster sampling, and its population composition as well as the villagers’ lifestyles were similar to that of rural areas of Xi’an. The inclusion criteria were (1) over 40 years old, (2) willing to join, and (3) informed consent. The exclusion criteria were (1) suffered from AD and other dementias, cerebrovascular diseases and other neurological conditions that may influence cognitive function (such as epilepsy, central nervous system infections, Parkinsonism, intracranial trauma, or surgery), (2) suffered from the climacteric syndrome, (3) failed to complete the Pittsburgh sleep quality index (PSQI) and the Mini-Mental State Examination(MMSE) or not finishing the clinical investigation, (4) had incomplete data.

The Medical Ethics Committee of the First Affiliated Hospital of Xi’an Jiaotong University approved this study, and informed consent was obtained from every participant. The enrolled subjects were first given a face-to-face structured interview for health status, medical history, and physical examination at the committee office or their own home if they had difficulties going to the office. Meanwhile, fasting blood samples of individuals were collected from 9 am to 11 am.

### Cognitive assessment

All the subjects were carried out a cognitive screening using the Mini-Mental State Examination (MMSE). The cutoff values were as follows: ≤17 for subjects with illiteracy, ≤ 20 for primary school educated subjects, and ≤ 24 for those educated at the junior high school level or above [21]. Participants with MMSE score ≤ cutoff values underwent the 2nd phase cognitive examinations, including the Fuld Object Memory Evaluation test, Rapid Verbal Retrieval test, Trail-Making test, Digit Span test, and Block Design test. The diagnosis of MCI was determined according to the diagnostic criteria for MCI [22] by a senior neurologist.

### Assesses of sleep quality

All subjects were asked to assess their sleep quality over a 1-month time interval by independently completing the Pittsburgh Sleep Quality Index (PSQI). PSQI is a self-rated questionnaire consisting of 8 parts(subjective sleep quality, wake-up time, sleep latency, sleep duration, habitual sleep efficiency, sleep interference, use of sleeping medication, and daytime dysfunction)and is obtained through 19 independent questions [23]. Sleep efficiency is the ratio of the total time spent asleep (sleep duration) in a night compared to the total amount of time spent in bed. Sleep disturbance was defined as a PSQI score > 5 [23].

### Other covariates

Participants self-reported sociodemographic variables (age, gender, and education level) and specific clinical factors (smoking and/or alcohol status), and disease variables (diabetes, hypertension, hyperlipidemia, anxiety, and depression). Participants also had a neurological examination, a systemic physical examination, blood biochemical examination. Body mass index (BMI) was calculated as weight divided by height squared and divided into four levels: underweight (BMI, < 18.5), normal weight (BMI, 18.5–23.9), overweight (BMI, 24-27.9), obese (BMI > 28). Smokers are defined as those who have smoked continuously or accumulated for 6 months or more during their lifetime [24]. Drinking is defined as exceeding a certain daily drinking amount (e.g. 3 standard cups per day) or drinking amount per time (e.g. 5 standard cups per time, at least once a week). The diagnoses of diabetes, hypertension, hyperlipidemia, anxiety, and depression were based on self-reported disease history or taking anti-hypertensive, anti-diabetic, lipid-lowering drugs, anti-depressants, or anti-anxiety medications, or newly diagnosed according to guidelines [25, 26].

### Statistical analysis

Histogram and normal probability plot of standardized residual, and residual plots were performed to ensure the normality and homogeneity of residuals. A comparison of approximately normal continuous variables was conducted by independent *t*-test and mean (SD). Mann-Whitney U test and median (interquartile range) were employed to compare the difference of the abnormal distributed continuous variables, and the χ^2^ test and number (percentage) were used to compare categorical variables.

The relationship between cognitive impairment and sleep disturbance was analyzed by simple Spearman’s correlation analysis first. Then, bivariate Logistic regression analysis was employed to adjust the confounding factors such as sociodemographic variables (age, gender, and education level), specific clinical factors (smoking and/or alcohol status, BMI), and disease variables (diabetes, hypertension, hyperlipidemia, anxiety and depression).

Next, using the same method to analyze the relationship between specific components of sleep disorder and MCI. All the analyses are two-sided. P < 0.05 was statistically significant. All statistical analyses were carried out using SPSS (IBM, USA,22.0 version). All of the graphs were drawn in GraphPad Prism (Texas,8.3.0 version).

## Results

### The characteristics of the Study Population

**Fig. 1:**
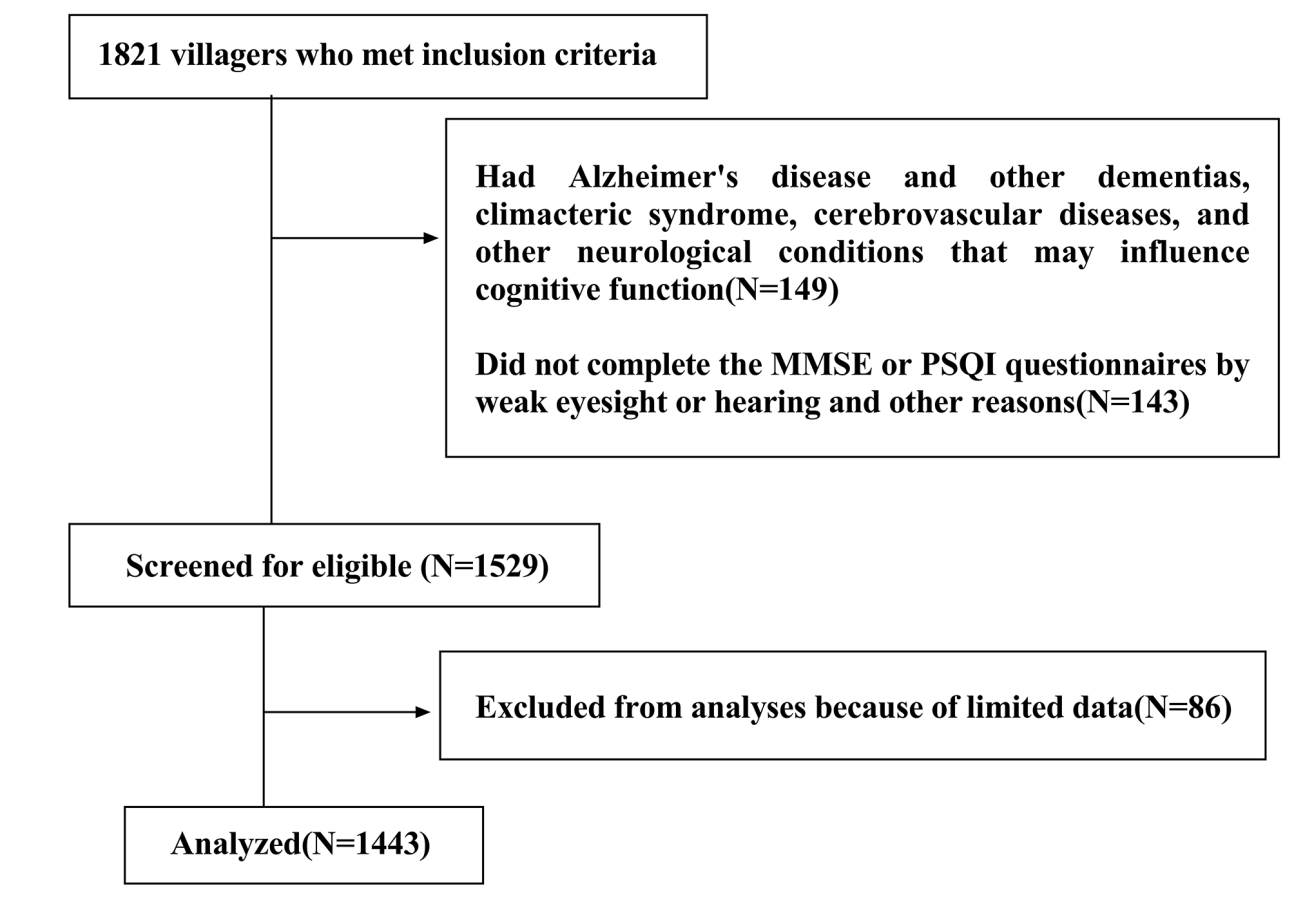
Flow chart of participant selection

1,821 villagers met the inclusion criteria. Of these, 149 had Alzheimer’s disease and other dementias, climacteric syndrome, cerebrovascular diseases, and other neurological conditions that may influence cognitive function, 143 did not complete questionnaires, and 86 were excluded because of limited data. Finally, 1,443 were included in the analysis (Fig. 1).

Among 1,443 participants, 830(57.52%) had sleep disturbance, and 69(4.78%) had cognitive impairment. Table 1 lists the clinical characteristics of the population. The total population is divided into normal sleep group and sleep disturbance, according to sleep quality. Compared with the normal sleep group, the sleep disturbance group was older, had more female subjects, shorter years of education, and had higher rates of drinking, hypertension, anxiety and depression but lower rates of smoking. The prevalence of MCI in the sleep disturbance group was higher than that in the normal sleep group (6.50% vs. 2.45%, *P* < 0.001).

**Table 1:** Characteristics of the total study population

Characteristics	Total (n = 1443)	Sleep disturbance group (n = 830)	Normal Sleep Group (n = 613)	P value
Age, years	57.02(9.51)	58.59(9.62)	54.90(8.95)	< 0.001
>60, n(%)	530(36.68)	357(43.01)	173(28.22)	< 0.001
Male, n(%)	583(40.35)	306(36.87)	277(45.19)	0.001
Education, years	6.79(6.84)	6.25(6.58)	7.54(7.12)	< 0.001
Drink, n(%)	467(32.32)	289 (34.82)	178(29.04)	0.020
Smoke, n(%)	444(30.73)	235(28.31)	209(34.10)	0.019
BMI, kg/m^2^	25.26(3.67)	25.28(4.04)	25.23(3.11)	0.822
Anxiety and depression, n(%)	61(4.22)	48(5.78)	13(2.12)	0.001
Hyperlipidemia, n(%)	286(19.79)	167(20.12)	119(19.41)	0.739
Diabetes, n(%)	162(11.21)	90 (10.84)	72(11.75)	0.592
Hypertension, n(%)	350(24.22)	235(28.31)	115(18.76)	< 0.001
Mild Cognitive Impairment, n(%)	69(4.78)	54(6.50)	15(2.45)	< 0.001

BMI, body mass index

### The factors Associated with mild cognitive impairment

According to the cognitive states, the total population is divided into the normal cognition group and the MCI group. Compared with the normal cognition group, the MCI group was older, had more female subjects, shorter years of education, and fewer smoking problems. Above all, the prevalence of sleep disturbance in the MCI group was higher than that in the normal sleep group (78.26% vs. 56.48%, *P* < 0.001) (Table 2).

**Table 2:** Comparison of sleep disturbance group and normal sleep group

Characteristics	Mild Cognitive Impairment group (n = 69)	Normal cognition group (n = 1374)	P value
Age, years	66.75(9.22)	56.54(9.27)	< 0.001
> 60, n(%)	50(72.46)	480(34.93)	< 0.001
Male, n(%)	20(28.99)	563(40.98)	0.048
Education, years	2.32 (3.27)	7.02(6.90)	< 0.001
Drink, n(%)	25(36.23)	442(32.17)	0.481
Smoking, n(%)	13(18.84)	431(31.37)	0.028
BMI, kg/m^2^	24.51(3.45)	25.30(3.68)	0.083
Anxiety and depression, n(%)	2(2.90)	59(4.29)	0.574
Hyperlipidemia, n(%)	13(18.84)	273(19.87)	0.834
Diabetes, n(%)	6(8.70)	156(11.35)	0.495
Hypertension, n(%)	22(31.88)	328(23.87)	0.130
Sleep disturbance, n(%)	54(78.26)	776(56.48)	< 0.001

BMI, body mass index

### Multiple analysis of factors associated with mild cognitive impairment

To clarify the variables related to MCI, a Spearman binary correlation analysis was conducted. MCI was positively correlated with sleep disturbance, and age, but negatively with education level, gender, and smoking (Table 3).

To remove the disturbance of confounding factors, binary logistic regression analysis was performed. MCI was positively associated with sleep disturbance (OR = 2.027, 95%CI = 1.112–3.698, *P *= 0.021) and age > 60 (OR = 2.760, 95%CI = 1.472–5.176, *P* = 0.002). Besides, MCI was negatively associated with elementary school education level (OR = 0.382, 95%CI = 0.190–0.768, *P* = 0.007), compared with illiterate **(**Table 4**)**.

**Table 3:** Correlation analysis between cognitive impairment and clinical basic variables

Variables	Mild Cognition Impairment	
	ρ	P
Sleep disturbance	0.094	< 0.001
Age > 60	0.166	< 0.001
Gender	-0.052	0.048
Education level	-0.150	< 0.001
Drinking	0.019	0.482
Smoking	-0.058	0.028
BMI	-0.047	0.074
Anxiety and depression	-0.015	0.574
Hyperlipidemia	-0.006	0.835
Diabetes	-0.018	0.495
Hypertension	0.040	0.130

BMI, body mass index

**Table 4:** Binary logistic regression of Mild Cognitive Impairment and clinical basic variables

Independent variables	P value	OR (95% CI)
Age > 60	0.002	2.760(1.472, 5.176)
Gender: male	0.965	1.019 (0.444, 2.337)
Elementary school	0.007	0.382 (0.190, 0.768)
Junior High school and above	0.101	0.399(0.133, 1.195)
Drinking	0.889	1.042(0.585, 1.857)
Smoking	0.274	0.595(0.235, 1.509)
BMI	0.137	0.944(0.875, 1.018)
Anxiety and depression	0.388	0.527(0.123, 2.255)
Hyperlipidemia	0.872	1.055 (0.548, 2.033)
Diabetes	0.754	0.868(0.358,2.103)
Hypertension	0.857	1.053(0.601, 1.845)
Sleep disturbance	0.021	2.027(1.112, 3.698)

BMI, body mass index

### The relationship between sleep disturbance and cognitive impairment

Compared to the normal cognitive group, the MCI group had a shorter sleep duration (5.5 h vs. 6.5 h, *P* = 0.002), lower habitual sleep efficiency (66.67% vs. 78.95%, *P*<0.001), and more sleep interference (6.0 points vs. 5.0 points, *P* = 0.003) (Table 5).

**Table 5:** Comparison of sleeping status in Mild Cognitive Impairment group and normal cognition group

Sleeping status	Mild Cognitive Impairment group	Normal cognition group	P value
Subjective sleep quality, point	1.00(1.00,1.00)	1.00(1.00,1.00)	0.966
Wake-up time, point	1.00(1.00,2.00)	1.00(1.00,2.00)	0.071
Sleep latency, min	30.00(10.00,60.00)	30.00(10.00,30.00)	0.970
Sleep duration, hour	5.50(4.25,7.25)	6.50(5.50,7.50)	0.002
Habitual sleep efficiency, %	66.67 (47.49,78.89)	78.95 (64.71,90.00)	< 0.001
Sleep interference, point	6.00(3.50,10.00)	5.00(3.00,8.00)	0.003
Use of sleeping medication, point	0.00(0.00,0.00)	0.00(0.00,0.00)	0.604
Daytime dysfunction, point	0.00(0.00,2.00)	0.00(0.00,1.00)	0.279
Total scores, point	8.00(6.00,11.00)	6.00(4.00,9.00)	< 0.001

As shown in Table 6, MCI was positively correlated with Sleep interference (ρ = 0.077, *P* = 0.003), but negatively with Sleep duration (ρ=-0.082, *P* = 0.002), Wake-up time (ρ=-0.085, *P* = 0.001) and Habitual sleep efficiency (ρ=-0.133, *P* < 0.001).

**Table 6:** Correlation analysis of Mild Cognitive Impairment and internal components of sleep disturbance

Sleeping status	Mild Cognition Impairment	
	ρ	P
Subjective sleep quality, point	0.001	0.966
Wake-up time, point	-0.085	0.001
Sleep latency, min	0.001	0.970
Sleep duration, hour	-0.082	0.002
Habitual sleep efficiency, %	-0.133	< 0.001
Sleep interference, point	0.077	0.003
Use of sleeping medication, point	0.014	0.605
Daytime dysfunction, point	0.028	0.279
Total scores	0.106	< 0.001

**Fig. 2:**
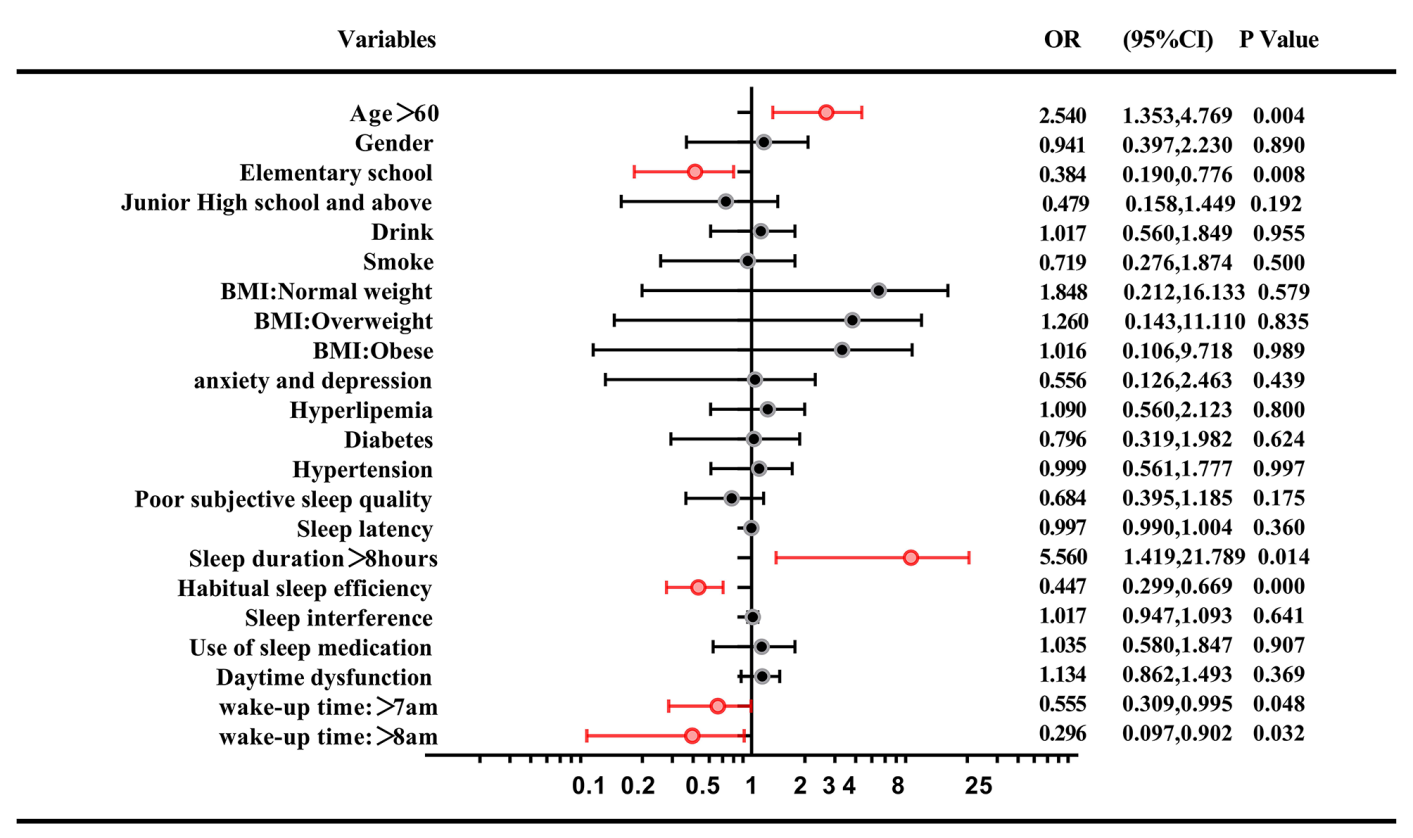
Logistic regression analyses of participant characteristics associated with the MCI BMI, body mass index. Statistically significant p-values are shown in red

To further exclude the interference of other factors, the internal composition of sleep and other basic variables were included in the binary logistic regression. As shown in **Fig. **2, MCI was still positively associated with the age>60 years old(OR = 2.540,95%CI = 1.353–4.769, *P* = 0.004), and negatively associated with the habitual sleep efficiency (OR = 0.447, 95%CI = 0.299–0.669, *P* < 0.001). MCI was negatively associated with waking up after 7 am (OR = 0.555, 95%CI = 0.309–0.995, *P* = 0.048), or 8 am (OR = 0.296, 95%CI = 0.097–0.902, *P* = 0.032), compared with waking up before or at 7 am. Besides, MCI was negatively associated with elementary school education level (OR = 0.384, 95%CI = 0.190–0.776, *P* = 0.008), compared with illiterate. In order to more accurately evaluate the effect of actual sleep duration on cognitive impairment, different sleep duration was included in the regression analysis in turn. It was found that MCI was positively associated with sleep duration of more than eight hours (OR = 5.560, 95%CI = 1.419–21.789, *P* = 0.014).

## Discussion

In this population-based cross-sectional study, we used MMSE to assess cognitive function and used PSQI to evaluate sleep quality in more than 1,400 subjects. We found that MCI was positively associated with the age>60 years old, and negatively associated with the habitual sleep efficiency. People who slept more than 8 h a day might be more likely to suffer from cognitive impairment. Having an education level above illiteracy may reduce the risk of MCI. Moderately waking up later probably is beneficial to maintain normal cognitive function, which is different from what we previously thought, compared to waking up too early.

Compared to previous studies, the present study is a large sample, community population-based study. We used a method of random cluster sampling to select villages, and the population composition was similar to that in the rural areas of Xi’an which make the sample reasonably represent the rural areas of Xi’an. The diagnosis of cognitive impairment was made according to the MMSE score first, and then the subjects underwent the 2nd phase of cognitive examinations, to make sure the diagnosis of MCI reliable. Meanwhile, we used PSQI to assess sleep quality over a 1-month time interval. PQSI is a self-rated questionnaire, which assesses sleep quality and disturbances. The scale consists of 8 parts(subjective sleep quality, wake-up time, sleep latency, sleep duration, habitual sleep efficiency, sleep disturbances, use of sleeping medication, and daytime dysfunction)and is obtained through 19 independent questions. PSQI score>5 yielded a diagnostic sensitivity of 89.6% and specificity of 86.5% (kappa = 0.75, *P*<0.001) for sleep disturbance [23]. Eight indicators have proven that PSQI to be a reliable and effective measure of insomnia, with a Cronbach’s α of 0.83 [23]. These assure that our results are reliable and convincing.

Similar findings were found that sleep duration is positively associated with MCI risk [27–29] [30], or in a U-shaped pattern [31–33]. This association between longer sleep duration and an increased risk of MCI was also observed in Hemodialysis patients and adults with metabolic syndrome [34, 35]. However, some study shows short sleep duration is a risk factor for MCI [36, 37], and there is a decreased risk of MCI for every additional hour of sleep among the elderly sleeping less than 7 h [38]. In this study, in order to more accurately evaluate the effect of actual sleep duration on cognitive impairment, different sleep duration was included in the regression analysis in turn, and after controlling for a variety of confounding factors, it was found that MCI may be more likely to occur if the sleep duration was more than eight hours.

Consistent with the findings of this study, several studies have suggested that habitual sleep efficiency is associated with MCI. Global cognitive performance is associated with sleep efficiency measured by polysomnography in patients with Parkinson’s disease [39]. Compared to never reporting sleep difficulty, difficulty initiating sleep most nights was associated with worse cognitive function over time as was difficulty waking up too early most nights [40]. Subjects with cognitive decline exhibited lower sleep efficiency, and reduced inter-hemispheric connectivity during rapid eye movement sleep [41].

There are few studies on the relationship between wake-up time and MCI, which is a gap in sleep and cognition research. This study found that people who woke up at or after 7 am perhaps had a lower risk of MCI.

Although the exact mechanism of sleep disturbance involved in cognitive impairment has not been fully determined. A growing number of studies have found that sleep disorders may lead to cognitive impairment and accelerate AD pathology, including increased Aβ deposition in the brain [42–48]. Our recent studies also showed that sleep deprivation induced peripheral Aβ transport dysfunction in young adults, and Aβ accumulation in the brain in rats [49, 50]. The underlying mechanism may involve the oxidative stress and inflammation [51, 52].

### Strengths

Compared with other studies with smaller sample sizes, this study was a large cross-sectional study with a sufficient study population and a well representative population. We excluded 149 individuals who suffered from AD and other dementias, cerebrovascular diseases, climacteric syndrome, and other neurological conditions (such as epilepsy, central nervous system infections, Parkinsonism, intracranial trauma, or surgery) that may influence cognitive function. Moreover, we adjusted our analyses for other confounders, such as sociodemographic variables (age, gender, and education level), specific clinical factors (smoking and/or alcohol status), and disease variables (diabetes, hypertension, hyperlipidemia, anxiety and depression).

In this study, sleep quality was evaluated by using the Pittsburgh sleep quality index (PSQI), which covers many aspects of sleep from subjective sleep quality, wake-up time, sleep latency, sleep duration, habitual sleep efficiency, sleep interference, use of sleeping medication, and daytime dysfunction. Assessing sleep quality with PSQI is more convenient and cost-effective, reflecting the usual sleep habits and quality without interference with the environment, thus allowing for assessing sleep quality in a large-sized study. Compared with the objective assessment of sleep quality, which was limited to a single night’s sleep, PSQI assesses sleep disturbances over a 1-month time interval.

The accuracy and reliability of the dependent variables were confirmed by the use of multiple cognitive scales and the final diagnosis of cognitive status by an experienced neurologist. Multiple covariates were included in binary logistic regression to eliminate the interference of numerous confounding factors to ensure the accuracy of our findings.

This study found that waking up before 7 am may be associated with an increased risk of MCI, which few studies have focused on before. In addition, this study also found that age over 60 years, illiteracy, lower sleep efficiency, and sleeping more than 8 h at night were associated with a higher risk of MCI. These findings will have important implications for the intervention of the risk of MCI and the prevention of dementia.

### Limitations

Due to cross-sectional design, which prevents us from clarifying any causal and temporal relationship between cognitive impairment and sleep disturbance. A follow-up study to detect the changes in cognition function is necessary. On the other hand, we did not use any sleep monitoring devices to objectively assess sleep conditions, such as polysomnography and activity tracking, but relied on self-reporting, which may cause recall bias. However, studies have shown that the use of polysomnography to record the results of sleep in the elderly is consistent with subjective reports [53]. In healthy people, the relationship between objective measures of sleep and “quality” sleep experience is not as strong as we might expect [54]. And people with mild cognitive impairment may not be able to describe their sleep with this questionnaire as accurately as normal cognitive people because of memory loss and other cognitive difficulties. In addition, polysomnography monitors are expensive and labor-intensive, which are not suitable for use in a large-sample epidemiological studies.

## Conclusion

This large-scale survey of 1,443 villagers found that sleep disturbance is associated with MCI, especially for sleep duration, wake-up time, and habitual sleep efficiency. However, the causal relationships between sleep disturbances and cognitive impairment are not clear, which need to be further studied.

## Data Availability

The datasets generated and analyzed during the current study are not publicly available due to privacy and ethical restrictions but are available from the corresponding author upon reasonable request.

## List of abbreviations

AD: Alzheimer’s disease
Aβ: β-amyloid
PSQI: Pittsburgh Sleep Quality Index
MMSE: Mini-Mental State Examination
MCI: Mild Cognitive Impairment
BMI: Body Mass Index
OSA: Obstructive Sleep Apnea
PS: Polygenic Score
